# Reduced Oligodendrocyte Precursor Cell Impairs Astrocytic Development in Early Life Stress

**DOI:** 10.1002/advs.202101181

**Published:** 2021-06-21

**Authors:** Yuxin Wang, Yixun Su, Guangdan Yu, Xiaorui Wang, Xiaoying Chen, Bin Yu, Yijun Cheng, Rui Li, Juan C. Sáez, Chenju Yi, Lan Xiao, Jianqin Niu

**Affiliations:** ^1^ Department of Histology and Embryology Chongqing Key Laboratory of Neurobiology Brain and Intelligence Research Key Laboratory of Chongqing Education Commission Third Military Medical University Chongqing 400038 China; ^2^ Research Centre Seventh Affiliated Hospital of Sun Yat‐sen University Shenzhen 518107 China; ^3^ Instituto de Neurociencia Centro Interdisciplinario de Neurociencia de Valparaíso Valparaíso 2381850 Chile; ^4^ Department of Neurosurgery 2nd affiliated Hospital Third Military Medical University Chongqing 400038 China

**Keywords:** astrocytic network, connexin, glial interaction, neuropsychiatric disorder, parental isolation

## Abstract

Astrocyte maldevelopment is implicated in various neuropsychiatric diseases associated with early life stress. However, the underlying astrocytopathy mechanism, which can result in the psychiatric symptoms, remains unclear. In this study, it is shown that a reduced oligodendrocyte precursor cell (OPC) population accompanies hindered hippocampal astrocytic development in an improved parental isolation mouse model, and that the loss of OPCs suppresses astrocytic network formation and activity. It is further demonstrated that OPC‐derived Wnt ligands, in particular Wnt7b, are required for Wnt/*β*‐catenin pathway‐mediated astrocytic development and subsequent effects related to neuronal function. In addition, focal replenishment of Wnt7a/b is sufficient to rescue astrocytic maldevelopment. These results elucidate a Wnt‐paracrine‐dependent but myelin‐independent role of OPCs in regulating astrocytic development, which provides a unique insight into the astrocytopathy mechanism in early life stress, and can be implicated in the pathogenesis of human early life stress‐related neuropsychiatric disorders.

## Graphical abstract

Astrocyte maldevelopment, including defective astrocytic network formation, is implicated in neuropsychiatric diseases induced by early life stress. It is shown that reduced oligodendrocyte precursor cell (OPC) number occurred in early life stress causes astrocyte maldevelopment through decreased level of OPC‐derived Wnt ligands, which subsequently elicits neuronal dysfunction and neuropsychiatric symptoms.

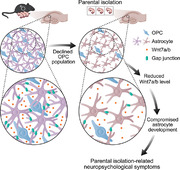

## 1 Introduction

With increasing and uneven urbanization in developing countries, the movement of migrant laborers has resulted in an increasing number of left‐behind children, who suffer from early life stress due to emotional or physical neglect.^[^ ^1^ ^]^ Early life stress is a major contributor to neuropsychiatric disorders in adulthood. Studies show that exposure to early life stress by these left‐behind children is directly implicated in psychiatric disorders such as emotional and psychosocial symptoms, including inclinations to impulsive behavior, anxiety, depression, and others.^[^ ^2^ ^]^ Although the primary underlying mechanism remains unclear, it has been considered that the normal brain function is altered.

The normal function of the central nervous system (CNS) relies upon not only the neurons, but also the precise coordination of the glial cells.^[^ ^3^ ^]^ Astrocytes are the most abundant glial cell type in the CNS. Mature and functional astrocytes form astrocytic networks through connexin‐based gap junctions, allowing signal propagation among astrocytes and communication with neurons and other glial cells,^[^ ^4^ ^]^ supporting local neuronal activity,^[^ ^5^, ^6^ ^]^ and even governing subsequent behavioral outcomes.^[^ ^4^, ^6^, ^7^ ^]^ Astrocyte maldevelopment and dysfunction, including dysregulation of astrocytic connexins, have been identified in maternal isolation animal models that mimic the early life stress,^[^ ^8^ ^]^ as well as in postmortem tissue from related‐neuropsychiatric patients, such as in depression and schizophrenia.^[^ ^9^, ^10^, ^11^ ^]^ However, the mechanism of astrocytopathies in early life stress‐induced neuropsychiatric disorders remains to be elucidated.

Intriguingly, insights from human postmortem brain tissues have shown that altered astrocytes are often associated with aberrant oligodendroglial lineage cells in neuropsychiatric disorders, such as major depressive disorder and schizophrenia,^[^ ^10^, ^11^, ^12^, ^13^, ^14^ ^]^ raising the possibility that these two different glial cell types may interact in the pathogenesis of neuropsychiatric disorders. Oligodendrocyte precursor cells (OPCs) are ubiquitously distributed throughout the CNS, even in the regions with sparse myelination.^[^ ^15^ ^]^ They can not only function as the precursors for myelinating oligodendrocytes (OLs), but also play a myelin‐independent role in maintaining brain function.^[^ ^16^, ^17^ ^]^ Previous studies have indicated that defective astrocytes can strongly impair OPC development and function in neural disorders,^[^ ^18^ ^]^ but whether this interaction can take place in the opposite manner during the progress of early life stress and contribute to the pathogenesis of neuropsychiatric disorders is still unknown.

In this study, we improved the traditional maternal isolation model by excluding the effects of nutrient‐supply difference, and assessed the interaction between astrocytes and OPCs. We found that reduced OPC‐derived Wnt ligands, in particular Wnt7b, are crucial for astrocytic maldevelopment leading to defective astrocytic network formation and activity. This process subsequently leads to neuronal dysfunction and neuropsychiatric symptoms. Our results demonstrate a new interaction manner between OPCs and astrocytes via the Wnt signaling pathway, and propose a novel etiological mechanism and a potential therapeutic target for early life stress‐related neuropsychiatric disorders.

## 2 Results

### 2.1 Improved Postnatal Parental‐Isolation Model Induced Neuropsychiatric Behaviors and Astrocyte Maldevelopment

**Figure 1:**
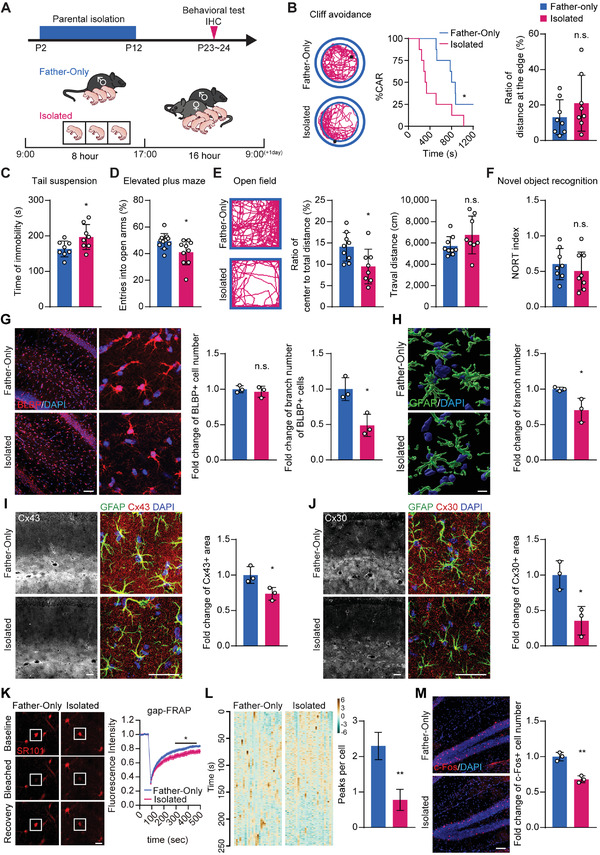
Improved parental isolation‐induced neuropsychiatric behaviors and astrocyte maldevelopment. A) To set up the isolated group (Isolated), mouse pups were separated from their parents and housed individually for 8 h daily (9:00–17:00) from P2 to P12. In the control group (Father‐only), mouse pups were separated from their mothers but stayed with their fathers. Mouse pups were subjected to behavioral tests and IHC analysis on P23. B) Cliff avoidance test: Left: representative diagram of the cliff avoidance test. Black dots represent the mouse position at the experiment endpoint. Middle: reduced cliff avoidance reaction (CAR) was observed in the isolated mice compared to the Father‐only control, suggesting an impulsive behavioral trait. Right: the ratio of travel distance at the edge to total distance was not significantly different between the two groups. The log‐rank (Mantel‐Cox) test was used to compare survival curves. C) Tail suspension test: Isolated mice displayed prolonged immobility times, suggesting depressive behavior. D) Elevated plus maze test: Isolated mice showed reduced entries into the open arm in the elevated plus maze test, suggesting anxiety‐like behavior. E) Open field test: Left: representative diagram of the open field test. The isolated mice exhibited a preference for staying at the edges. Middle and right: the open field test showed that isolated mice traveled less distance and spent less time in the central area, while they showed no statistical differences in mobility, as suggested by travel distance measurements. F) Novel object recognition test: no statistical differences in cognitive function between the Father‐only control and isolated mice. G) BLBP staining showed no difference in astrocyte numbers, but instead displayed reduced astrocyte processes per cell in isolated mice. Scale bar: 100 µm. H) 3D reconstruction of GFAP staining. GFAP^+^ branch number was reduced in isolated mice. Scale bar: 10 µm. I) Immunostaining of GFAP and astrocytic network proteins Cx43. Reduced Cx43^+^ area was found in isolated mice. Scale bar: 50 µm. J) Immunostaining of GFAP and astrocytic network proteins Cx30. Reduced Cx30^+^ area was found in isolated mice. Scale bar: 50 µm. K) gap‐FRAP: Left: representative images of the gap‐FRAP experiment at three different time points. Right: gap‐FRAP experiments showed that the fluorescence recovery rate and amplitude were decreased in the astrocytes of the isolated group, suggesting reduced gap junction channel function. Scale bar: 20 µm. Data presented as mean ± SEM, *n* = 10. L) Calcium Imaging on acute brain slices stained with Rhod‐4 showed that Isolated mice displayed reduced astrocytic Ca^2+^ waves. Data presented as mean ± SEM, *n* = 27. M) The isolated mice demonstrated fewer c‐Fos^+^ neurons. Scale bar: 50 µm. Data presented as mean ± SD unless stated otherwise. *N* ≥ 7 mice for behavioral tests, *N* = 3 mice for immunostaining experiments. *p*‐values are calculated using unpaired *t*‐test. *p < 0.05, **p < 0.01, n.s. not significant.

Traditional postnatal maternal‐isolation models are established by separating mouse pups from their mothers and littermates. However, these models suffer from a significant caveat, as control groups not only have regular caring but also access to milk feed.^[^ ^19^ ^]^ To distinguish the effects caused by the lack of caring from nutrient deficiency, we established a new control group, in which mouse pups were separated from their mothers but remained with their fathers, who can provide parental care^[^ ^20^ ^]^ but not milk (**Figure** **1**A). Our results mimicked the neuropsychological symptoms due to the lack of parenting, showing that the isolated mouse displayed significantly higher rates of impulsive (Figure 1B), depressive (Figure 1C), and anxiety‐associated (Figure 1D,E) behaviors but no cognitive dissonances (Figure 1F).

We then sought to examine the histological and functional changes in the astrocytes. Astrocytic features were significantly altered in the hippocampus, displaying more prominent histological alterations (Figure S1A, Supporting Information), including astrocyte branches reduction (reduced to 48.9% shown by BLBP staining, Figure 1G, and 70.4% by GFAP, Figure 1H) and downregulation of astrocytic network markers Cx43 and Cx30 (73.5% and 35.5% of the control, respectively, Figure 1I,J), while the total number of astrocytes were not different (Figure 1G). The astrocytic network dysfunction could be demonstrated by compromised astrocytic gap junction communication. As shown in gap‐FRAP experiments,^[^ ^21^ ^]^ fluorescence recovery after photobleaching was slower in the isolation group when compared to the Father‐only group (Figure 1K). Additionally, calcium imaging showed that the intracellular Ca^2+^ activity in astrocytes was downregulated in the isolation group, indicating astrocytic activity was suppressed (Figure 1L).

As neuronal activity is closely associated with and governed by astrocyte morphology and function,^[^ ^22^ ^]^ we observed that isolated mice displayed a repressed neuronal activation shown by 34.7% lower c‐Fos level in the hippocampus, an important encephalic region associated with the emotional and psychological behavioral changes,^[^ ^23^ ^]^ albeit with an unaltered neuron number (Figure 1M and Figure S1B, Supporting Information).

Together, our model indicates that the astrocyte maldevelopment, such as defective astrocytic network and repressed activity, is a major pathological feature of the parentally isolated mice, which is a critical factor in altering subsequent neuronal activity and neuropsychiatric behaviors.

### 2.2 OPC Population Reduction Induced Astrocyte Maldevelopment in a Paracrine Manner

**Figure 2:**
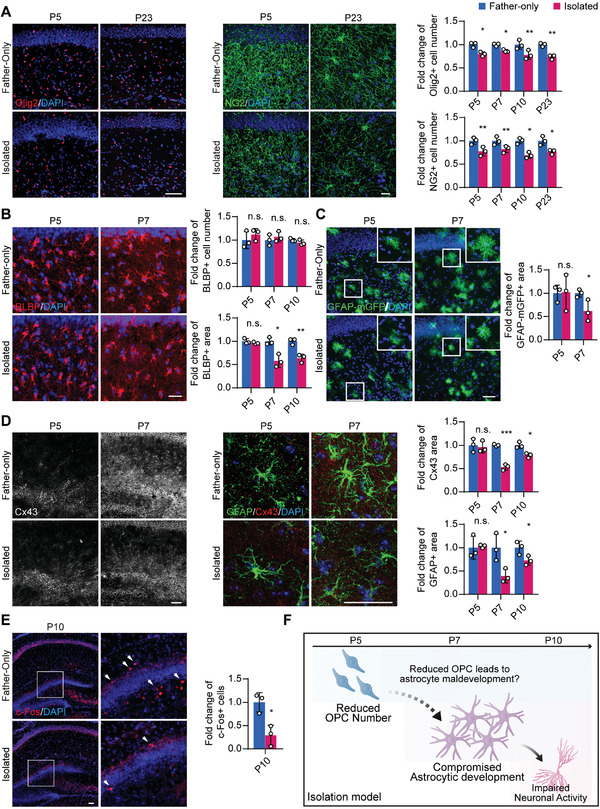
OPC population decrease precedes astrocytic and neuronal abnormalities in the isolation model. A) The number of Olig2 or NG2‐labeled oligodendroglial lineage cells was reduced in the isolation group as early as P5. Scale bar: 100 µm in Olig2 staining, 20 µm in NG2 staining. B) BLBP staining revealed that the BLBP^+^ area of the isolated group started to decrease on P7 compared to the control. Scale bar: 20 µm. C) GFAP promoter‐dependent membrane GFP expression was used to determine the astrocyte domain size, showing that the isolated group has decreased astrocyte size at P7 but not P5. Scale bar: 50 µm. D) Staining of GFAP and Cx43 showed that the decrease in the GFAP^+^ and Cx43^+^ levels also started to emerge on P7. Scale bar: 50 µm. E) The number of c‐Fos^+^ neurons was decreased in the isolated group at P10. Scale bar: 50 µm. F) The sequential occurrence of OPC and astrocyte abnormalities suggests the possible regulation of astrocyte development by OPCs, while astrocytic regulation of neuron activity has already been confirmed by prior studies.^[^ ^4^, ^6^, ^7^ ^]^ Data presented as mean ± SD, *N* = 3 mice. *p*‐values are calculated using unpaired *t*‐test. *p < 0.05, **p < 0.01, ***p < 0.001, n.s. not significant.

We next questioned how astrocytes are affected in the pathogenesis of our model, and whether oligodendroglial lineage cells were implicated in astrocytic pathogenesis. We found that oligodendroglial lineage cells were altered in the hippocampus and cortex, the same regions where astrocytes are found to be changed (Figure S1A, Supporting Information). In the isolation group, the total number of hippocampal OPCs was significantly reduced (76.5% by Olig2 staining and 73.8% by NG2) in the hippocampus (**Figure** **2**A). When chronologically traced these histological abnormalities in parentally isolated mice at earlier stages, we further revealed that this reduction of OPC number took place as early as P4, which could be attributed to reduced OPC proliferation instead of increased cell death (Figure S1C,D, Supporting Information). In addition, the differentiation capacity of OPCs remained intact, as we found that the proportion of CC1^+^/Olig2^+^ mature OLs and the structures of MBP^+^ myelin were unchanged (Figure S1E,F, Supporting Information). As early as P7, astrocytic features started to display a shrunken morphology, including shortened BLBP^+^, GFAP‐mGFP^+^, and GFAP^+^ branches and decreased immunoreactivity areas (Figure 2B–D), which were also accompanied by downregulation in the astrocytic network marker Cx43 (Figure 2D). In addition, a decrease of neuronal activity as shown by c‐Fos level in the hippocampus was detected until P10 (Figure 2E), while the numbers and morphologies of both neurons and microglia were not altered (Figure S1G,H, Supporting Information). The sequential occurrence of OPC and astrocyte abnormalities led us to hypothesize that reduction of OPCs may trigger astrocytic maldevelopment in parentally isolated mice (Figure 2F).

**Figure 3:**
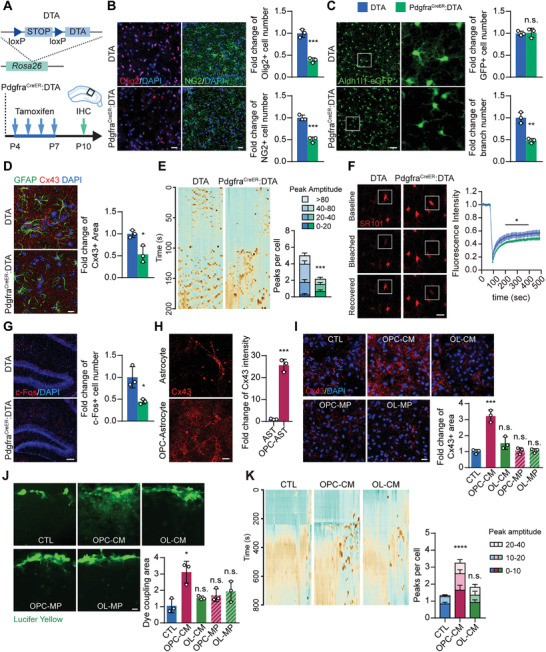
OPC population reduction‐induced astrocyte maldevelopment. A) DTA mouse genetic construction and Tamoxifen administration. DTA mice were mated with Pdgfra^CreER^ mice to produce Pdgfra^CreER^:DTA mice, which allows the depletion of OPCs by DTA expression after Tamoxifen treatment. Tamoxifen was administered to mice from P4‐P7; this was followed by IHC experiments at P10 to analyze histological changes in the hippocampus. B) Olig2 and NG2 staining confirmed efficient depletion of OPCs in the hippocampus of Pdgfra^CreER^:DTA mice. DTA, the control group, was set up using DTA only littermates. Scale bar: 20 µm. C) Aldh1l1‐eGFP mice were also crossed to the DTA mice, which showed that OPC ablation reduced GFP+ astrocyte branch number but not the number of GFP+ astrocytes. D) Immunostaining of GFAP and Cx43 showed that OPC‐ablated mice displayed reduced expression of Cx43. Scale bar: 10 µm. E) Calcium imaging on acute brain slices showed that the number of calcium peaks per astrocyte during the observation was reduced in Pdgfra^CreER^:DTA mice. This reduction was also observed in peak amplitude. Scale bar: 20 µm. Data presented as mean ± SEM, *n* = 32. F) gap‐FRAP experiments showed that the fluorescence recovery rate and amplitude were decreased in the astrocytes of Pdgfra^CreER^:DTA mice, suggesting reduced gap junction channel function. Scale bar: 20 µm. Data presented as mean ± SEM, *n* = 8. G) Immunostaining of c‐Fos showed reduced neuron activity in OPC‐ablated mouse hippocampus. Scale bar: 50 µm. H) OPC‐astrocyte co‐culture promoted the expression of Cx43 in astrocytes. Scale bar: 10 µm. I) Conditioned medium (CM) or isolated membrane proteins (MP) were obtained from OPC or OL culture, and were added to the primary astrocyte culture for 24 h. Only treatment of OPC‐CM was able to promote Cx43 expression in astrocytes. Scale bar: 20 µm. J) Scratch experiments showed that the dye coupling area was significantly increased by OPC‐CM treatment. Scale bar: 20 µm. K) Calcium imaging experiments showed that OPC‐CM treatment increased the calcium spikes in astrocytes but not OL‐CM treatment. Data presented as mean ± SEM, *n* = 45. Data presented as mean ± SD unless stated otherwise. *N* = 3 mice or 3 independent experiments. *p*‐values are calculated using unpaired *t*‐test or one‐way ANOVA. *p < 0.05, **p < 0.01, ***p < 0.001, n.s. not significant.

To test our hypothesis, we first generated OPC‐eliminated mice (Pdgfra^CreER^: DTA) (**Figure** **3**A) to investigate the impact of OPC elimination (Figure 3B) on astrocyte development in the hippocampus. Similar to that of parentally isolated mice, astrocytes in the Pdgfra^CreER^: DTA mice exhibited shrunken morphologies (Figure 3C), decreased expression of Cx43 (Figure 3D), along with abrogated gap junction channel function (Figure 3E) and reduced Ca^2+^ activity (Figure 3F). In addition, lower neuronal activity was also detected in OPC‐eliminated mice similar to that of our isolation model at P10 (Figure 3G), while the numbers and morphologies of neuron and microglia were not altered (Figure S2A,B, Supporting Information). To validate the specific oligodendroglial lineage targeting of the Pdgfra^CreER^ mice, we crossed them with the Rosa‐YFP reporter mice. These mice showed that YFP^+^ cells were rarely co‐labeled with neuronal marker (0.0450%) and no YFP^+^ cells were co‐labeled with astroglial marker (Figure S2C,D, Supporting Information), consistent with the previous report.^[^ ^24^ ^]^ In agreement with our in vivo results, astrocytes co‐cultured with OPCs expressed a higher level of network protein Cx43 (Figure 3H and Figure S2E, Supporting Information).

To examine whether this interaction took place in a paracrine or a direct‐contact manner, we treated primary cultured astrocytes with OPC‐ or OL‐derived conditioned medium (CM) or extracted membrane proteins (MP), and found that only OPC‐CM treatment was able to significantly increase Cx43 expression (Figure 3I), and was furthermore able to promote gap junctional communication (Figure 3J), and enhancement of Ca^2+^ activity in the astrocytes (Figure 3K). This suggests that OPCs promote astrocyte development and function in a paracrine manner.

Taken together, these results demonstrate that OPCs regulate astrocyte development in a paracrine manner, and reduction of the OPC population causes astrocyte maldevelopment in the parental isolation model.

### 2.3 Downregulation of Wnt/*β*‐Catenin Signaling Mediated Astrocyte Maldevelopment

**Figure 4:**
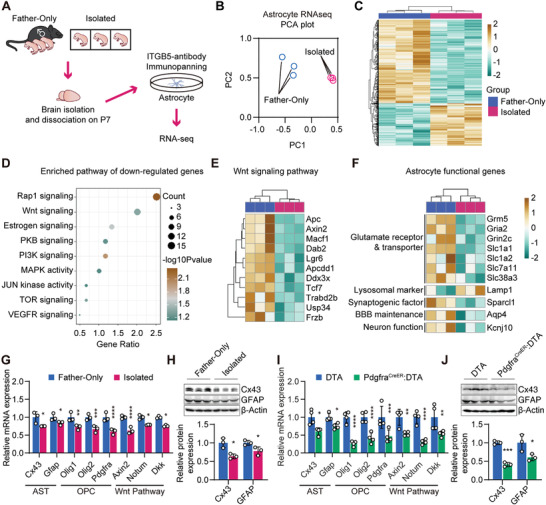
Downregulation of Wnt/*β*‐catenin signaling coupled with astrocyte maldevelopment. A) RNA‐seq experiments were conducted on acutely isolated astrocytes from the Father‐only group and the parentally isolated group at P7. B) Principal component analysis plot of astrocyte RNA‐seq data of the isolation model. C) Heatmap showing differentially expressed genes in astrocytes between the Father‐only group and the parentally isolated group. D) Functional annotation of genes downregulated in the isolated group. E) Differentially expressed genes within the Wnt pathway from RNA‐seq. F) Differentially expressed genes involve in astrocytic function.^[^ ^25^ ^]^ G) RT‐qPCR conducted on brains from Father‐only and the parentally isolated groups, revealed the decreases of astrocytic network markers, OPC population, and Wnt tone within the brain. H) Western blot experiment of the total hippocampal lysate of the isolation model confirmed the downregulation of Cx43 and GFAP expression. I) RT‐qPCR conducted on brains from the control (DTA) and Pdgfra^CreER^:DTA mice, revealed decreases of astrocytic network markers, OPC population, and Wnt tone within the brain. J) Western blot experiment confirmed the downregulation of Cx43 and GFAP expression. Data presented as mean ± SD. *N* = 3 mice in maternal isolation model, *N* = 4 mice in OPC‐ablated mouse model. *p*‐values are calculated using unpaired *t*‐test. *p < 0.05, **p < 0.01, ****p < 0.0001, n.s. not significant.

To further identify the underlying mechanism of how OPCs facilitate astrocytic development and which factors released from the OPC are involved in regulating astrocyte development, we used RNA‐seq to profile the transcriptome in astrocytes purified from P7 control and parental isolated mice (**Figure** **4**A–C). Pathway enrichment analysis of differentially expressed genes identified two highly related signaling pathways, Rap1 and Wnt (Figure 4D). The Wnt, but not the Rap1 pathway,^[^ ^26^ ^]^ has been reported to participate in astrocyte activation,^[^ ^27^ ^]^ and is also altered in abnormal astrocytes induced by stress.^[^ ^28^ ^]^ We further found that a series of Wnt/*β*‐catenin pathway related genes was downregulated, such as Axin2 and APC (Figure 4E), while several astrocyte function‐related genes were also downregulated, including Grm5, Slc1a2, Slc38a3, Sparcl1, Aqp4, and Kcnj10 (Figure 4F). Similarly, in both parental isolation and OPC‐ablation mice, we confirmed that the mRNA levels of astrocytic mature markers (*Cx43* and *Gfap*) and oligodendroglial lineage markers (*Olig1*, *Olig2*, and *Pdgfra*) were decreased, and were accompanied by the downregulation of the mRNA levels of Wnt/*β*‐catenin pathway related molecules (*Axin2*, *Notum*, and *Dkk1*) (Figure 4G,I). Reduced expression of Cx43 and GFAP in parental‐isolation and OPC ablation mice was further confirmed by Western blot (Figure 4H,J and Figure S3E, Supporting Information). Considering that Cx43, Kcnj10, Slc1a2, and Aqp4 have previously been shown to be regulated by Wnt/*β*‐catenin pathway in prior studies,^[^ ^29^ ^]^ our results suggest that astrocyte maldevelopment and dysfunction in parentally isolated mice are closely associated with the downregulation of the Wnt/*β*‐catenin pathway.

### 2.4 OPC‐Derived Wnt7a/b Were Essential for Astrocyte Wnt/*β*‐Catenin Pathway Activation and Development

We then attempted to understand how OPCs regulate astrocytic Wnt/*β*‐catenin pathway activation. OPCs have been recently identified as a source of Wnt ligands, such as Wnt7a and Wnt7b which are highly expressed.^[^ ^30^ ^]^ In our isolated mice, the expression of Wnt ligand in OPCs was unaltered (Figure S3A, Supporting Information), nor was the expression of Wnt receptors and ligands in astrocytes (Figure S3B, Supporting Information). Nonetheless, we found a reduction of the OPC population coupled with a decrease in Wnt7a/b expression in parentally isolated and OPC ablation mice (Figure S3C,D, Supporting Information). We hence speculated that downregulation of total amount Wnt ligands caused by reduced OPC population is responsible for downregulation of the Wnt/*β*‐catenin pathway and subsequent astrocyte maldevelopment in parental isolation mice.

**Figure 5:**
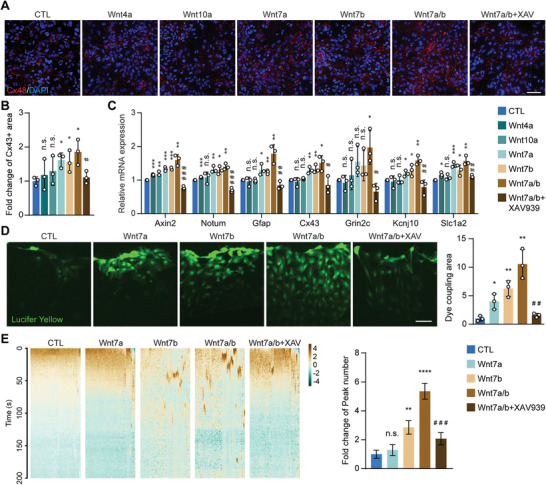
OPC‐derived Wnt ligands Wnt7a/b promote astrocytic network development in vitro. A,B) Wnt ligand‐treated primary astrocyte cultures were subjected to immunofluorescence staining of Cx43. Wnt7a and Wnt7b, individually or combined, were able to promote the expression of Cx43, and this effect was abolished by the Wnt signaling pathway inhibitor XAV939. Scale bar: 50 µm. C) Wnt ligand‐treated primary astrocyte cultures were subjected to RT‐qPCR to detect the expression of astrocyte functional genes. Wnt ligands, especially Wnt7a and Wnt7b, were able to promote the expression of Gfap, Cx43, Grin2c, Kcnj10, and Slc1a2, which was abolished by Wnt signaling pathway inhibitor XAV939. Axin2 and Notum were used as the positive control of Wnt signaling pathway activation. D) Wnt ligand‐treated primary astrocyte cultures were subjected to the scratch assay. Wnt7a and Wnt7b treatment, individually or combined, was able to increase the dye coupling area, and this effect was abolished by the Wnt signaling pathway inhibitor XAV939. Scale bar: 50 µm. E) Heatmap of calcium imaging in Wnt ligand‐treated primary astrocyte cultures. An increased number of Ca^2+^ wave peaks could be induced by Wnt7a/b treatment. Data presented as mean ± SEM, *n* = 68. Data presented as mean ± SD unless stated otherwise. *N* = 3 independent experiments. *p*‐values are calculated using one‐way ANOVA with multiple comparison test. *p < 0.05, **p < 0.01, ***p < 0.001, ****p < 0.0001, n.s. not significant. ^#^comparison between Wnt7a/b and Wnt7a/b+XAV939 groups. ^#^ *p* < 0.05, ^# #^ *p* < 0.01, ^# # #^ *p* < 0.001.

We set out to verify if OPC‐expressed Wnt ligands could promote astrocyte development in vitro. Cultured astrocytes were treated with a series of Wnt ligands, including Wnt4, Wnt10a, Wnt7a, Wnt7b, and Wnt7a plus Wnt7b (Wnt7a/b). We found that Wnt7a, Wnt7b, especially Wnt7a/b, upregulated astrocytic mature markers (*Cx43* and *Gfap*) (**Figure** **5**A–C), functional genes (*Grin2c*, *Kcnj10*, and *Slc1a2*), and Wnt/*β*‐catenin pathway molecules (*Axin2* and *Notum*) (Figure 5C), promoted the astrocytic network formation (Figure 5D), and enhanced Ca^2+^ activities in astrocytes (Figure 5E). In addition, XAV939, an antagonist of Wnt/*β*‐catenin signaling, blocked these effects of Wnt7a/b in astrocytes, indicating that astrocyte development can be regulated by OPC‐derived Wnt7a/b, and suppression of the Wnt/*β*‐catenin pathway represses astrocyte development and activity.

**Figure 6:**
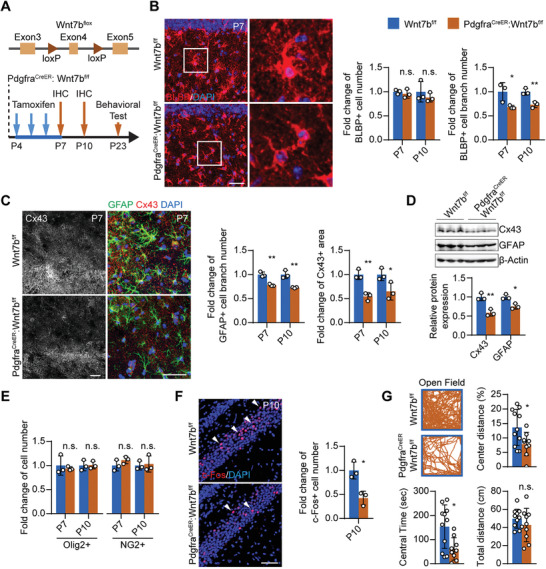
OPC‐derived Wnt ligand Wnt7b is essential for astrocytic network development and neurological function in vivo. A) Construction of Wnt7b‐flox mouse strain and the overall experiment scheme. B) Immunostaining of BLBP showed that Wnt7b cKO resulted in shrunk BLBP morphology but did not affect BLBP^+^ cell number at P7 and P10. C) Immunostaining of GFAP and Cx43 showed that Wnt7b cKO resulted in reduced astrocyte processes and astrocytic Cx43 expression. Scale bar: 50 µm. D) Western blot result of total hippocampal protein lysate confirmed the reduced Cx43 and GFAP expression in Wnt7b cKO. E) Immunostaining of Olig2 and NG2 reflected no significant change in the OPC number in the Wnt7b cKO. F) Immunostaining of c‐Fos showed that Wnt7b cKO resulted in decreased c‐Fos+ neurons. Scale bar: 100 µm. G) Open field experiments showed that Wnt7b cKO mice spent less time and traveled a shorter distance in the center area, while the total travel distance was not significantly altered. Data presented as mean ± SD unless stated otherwise. *N* ≥ 9 mice for behavioral tests, *N* = 3 mice for immunostaining experiments. *p*‐values are calculated using unpaired *t*‐test. *p < 0.05, **p < 0.01, n.s. not significant.

To further confirm the critical role of OPC‐derived Wnt7a and Wnt7b in astrocytes, we conditionally knocked out Wnt7b or Wnt7a in OPCs by crossing floxed/Wnt7b and floxed/Wnt7a with Pdgfra^CreER^ mice (referred to Wnt7b cKO and Wnt7a cKO, respectively) (**Figure** **6**A and Figure S5A, Supporting Information). In Wnt7b cKO mouse brain, we found significantly lower expression of Wnt7b, accompanied by reduced expression of astrocyte marker (*Gfap* and *Cx43*) (Figure S4A,B, Supporting Information). Consistent with our findings in parental isolated and OPC‐ablation mice, Wnt7b cKO mice also displayed diminished astrocyte branches; 68.0% of control by BLBP staining and 77.9% by GFAP staining (Figure 6A–C and Figure S4C,D, Supporting Information), and 55.5% lower expression levels of Cx43 compared to control at P7 (Figure 6C,D and Figure S4D,J, Supporting Information). Intriguingly, the neuronal activity, as shown by the c‐Fos level, was decreased (Figure 6F) without changes in the numbers of neurons (Figure S4H, Supporting Information), oligodendroglial cells (Figure 6E and Figure S5E–G, Supporting Information), or microglial cells (Figure S4I, Supporting Information). Furthermore, these mice displayed anxiety‐associated behaviors during the Open Field experiments (Figure 6G). However, no such phenotypes could be found in the Wnt7a cKO mice (Figure S5, Supporting Information). This may be partially explained by the fact that OPCs release significantly less Wnt7a as compared to Wnt7b (Figure S6A,B, Supporting Information).

Wnt7a and Wnt7b have also been reported to mediate brain endothelial cell and vasculature development and/or function,^[^ ^31^, ^32^, ^33^ ^]^ which may influence astrocyte development and neuronal function through insufficient oxygen and nutrition supplementations in this study. However, consistent with previous studies,^[^ ^31^, ^32^, ^33^ ^]^ we did not find changes in endothelial cell proliferation after OPC‐CM, Wnt7a (0.15 ng mL^−1^), or Wnt7b (0.45 ng mL^−1^) treatments (Figure S6C–E, Supporting Information), or differences in the vasculature morphology in the hippocampus of Wnt7a cKO and Wnt7b cKO mice (Figure S6F,G, Supporting Information). Based on our evidence which shows a normal vasculature network in both parental isolation and OPC‐ablation models (Figure S6H,I, Supporting Information), we excluded the possibility of endothelial dysfunction in our models.

Taken together, our results demonstrate that OPC‐derived Wnt7a/b, in particular Wnt7b, is essential for astrocytic Wnt/*β*‐catenin pathway activation, development, and function. The lack of OPC‐derived Wnt7b can consequently elicit neuronal dysfunction and neuropsychiatric symptoms.

### 2.5 Wnt7a/b Replenishment Rescued the Astrocytic Maldevelopment in Parental Isolation Mice

**Figure 7:**
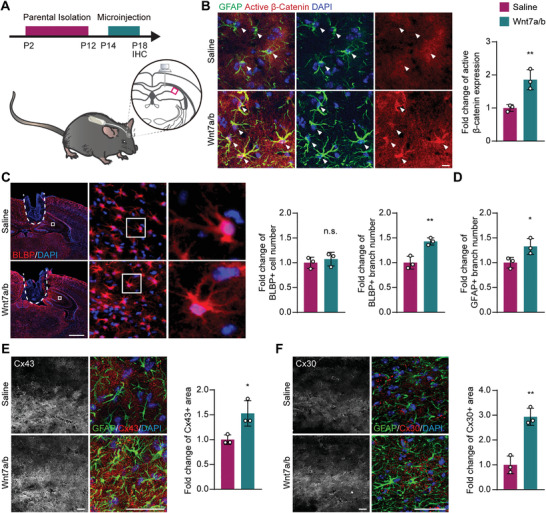
Wnt7a/b replenishment rescues the astrocytic network abnormality in the parental isolation model. A) Demonstration of micro‐injection procedure. The observation site was highlighted by the magenta square. B) Immunostaining of GFAP and active (nonphosphorylated) *β*‐catenin. Arrowheads highlight GFAP+ cells. Scale bar: 10 µm. GFAP+ astrocytes in Wnt7a and Wnt7b injected mice had a significantly higher level of active *β*‐catenin compared to 0.9% NaCl injected control (Saline). C) Immunostaining of BLBP showed that the number of BLBP^+^ cells was not altered by Wnt7a and Wnt7b injection, while the branch number was increased. The injection site was highlighted by the dashed line. Scale bar: 1000 µm. D) Wnt7a and Wnt7b injected mouse displayed increased GFAP^+^ cell branches. E,F) Immunostaining of Cx43, Cx30, and GFAP showed increased expression of Cx43, Cx30 in Wnt7a and Wnt7b injected mouse hippocampus. Scale bar: 50 µm. Data presented as mean ± SD. *N* = 3 mice for immunostaining experiments. *p*‐values are calculated using unpaired *t*‐test. *p < 0.05, **p < 0.01, n.s. not significant.

Based on this mechanism, we sought to examine whether the pharmacological intervention of Wnt/*β*‐catenin signaling could alleviate astrocytic abnormalities in parental isolation mice. We micro‐injected Wnt7a, Wnt7b, and Wnt7a/b into the hippocampus of the parental isolation mice at P14. After a 4 days replenishment period of these Wnt ligands, we analyzed the histological changes in the glial cells between 500 and 600 µm distal from the injection site (**Figure** **7**A). Micro‐injection of Wnt7a/b was sufficient to induce activation of Wnt/*β*‐catenin pathway in astrocytes, as shown by the elevated level of active *β*‐catenin immunostaining (Figure 7B). Additionally, Wnt7a/b micro‐injection was able to focally rescue astrocytic morphological immaturation (increased by 42.9% in BLBP^+^ branch number and 32.8% in GFAP^+^, Figure 7C,D) and significantly increase expression of the network proteins Cx43 and Cx30 (increased by 52.9% and 193.7%, respectively, Figure 7E,F). Consistent with our in vitro experiments, similar but milder astrocytic phenotypes were observed in Wnt7b micro‐injected parental isolation mice (Figure S7A, Supporting Information). However, the micro‐injection of Wnt7a, Wnt7b, or Wnt7a/b did not affect cell numbers of OPC (Figure S7B,C, Supporting Information) or microglia (Figure S7D, Supporting Information) when compared with the saline controls.

These results demonstrate that the astrocytic maldevelopment in parental isolation mice can be restored by the replenishment of Wnt7a/b, suggesting a possible therapeutic strategy for early life stress‐related neuropsychiatric disorders.

## 3 Discussion

Left‐behind children, who by definition lack parental care, display certain neuropsychiatric and behavioral disorders. A better understanding of the underlying mechanism, in particular how glial interactions play a part in neuropsychiatric disorders in this population is of critical interest. However, most mechanistic studies of disease focus only on a single cell type and ignore the interactions between different glial cell types, which severely limits our understanding of the pathogenesis of neuropsychiatric disorders. In this study, we demonstrate a novel OPC‐astrocyte crosstalk via the Wnt/*β*‐catenin signaling. This previously unidentified Wnt ligand‐dependent role of OPCs in regulating astrocytic development can be implicated in the pathogenesis of early life stress‐related neuropsychiatric disorders.

Astrocyte development is essential for normal neuronal function in the CNS by trafficking ions, neurotransmitters, glucose, and lactate to coordinate local neuronal activities. However, these functions are compromised under pathological conditions.^[^ ^34^ ^]^ A recent study revealed that stress impairs the astrocytic network by downregulating Cx43, and disrupts synaptic plasticity in the hippocampus and neocortex.^[^ ^28^ ^]^ In our study, we further demonstrated that the astrocyte maldevelopment in early life stress was mediated by the loss of OPCs. Increasing lines of evidence show that the OPC is not merely a passive “recipient” in the CNS, but also closely communicates with myelinating axons,^[^ ^35^ ^]^ T cells,^[^ ^16^ ^]^ and the brain vasculature.^[^ ^17^ ^]^ In the adult mouse brain, OPC depletion impairs astrocytic uptake of extracellular glutamate and triggers neurological symptoms.^[^ ^14^ ^]^ In our study, we discovered a novel paracrine‐mediated OPC regulatory mechanism of astrocyte development, suggesting a new role of OPCs in modulating brain function and diseases, which may explain previous findings that a reduced number of oligodendroglial lineage cells is associated with the altered astrocytic morphology in patients with mental disorders.^[^ ^10^, ^12^ ^]^

As reported previously, proper Wnt signaling is required for choreographed development and function of the CNS.^[^ ^36^ ^]^ The Frizzled family of Wnt ligand receptors are widely expressed in the brain with cell type‐specific inter‐family variety and widely differing expression levels. Specific receptors in the family possess a preference for different subsets of Wnt ligands, thereby enabling a mechanism of differential regulation between cell types.^[^ ^30^, ^37^ ^]^ For examples, Wnt7a and Wnt7b have been shown to be responsible for changes in OPCs,^[^ ^31^ ^]^ neurons,^[^ ^38^ ^]^ as well as brain vasculature.^[^ ^31^, ^32^, ^33^, ^39^ ^]^ Interestingly, previous studies report that neurons and endothelial cells respond to Wnt7a at a high concentration (50 ng mL^−1^ and 0.5 µg mL^−1^, respectively),^[^ ^33^, ^38^ ^]^ but we found that astrocytes can respond to a comparably lower concentration of exogenous Wnt7a (0.15 ng mL^−1^), and especially Wnt7b (0.45 ng mL^−1^). This suggests that astrocytes are more sensitive to Wnt7a and Wnt7b during development as compared to other brain cell types, and it would be more feasible to control astrocyte development by regulating Wnt/*β*‐catenin signaling. Indeed, the astrocytic maldevelopment in parental isolation mice was found to be restored by the replenishment of Wnt7a/b or partly restored by Wnt7b in our study. This indicates that the early dysfunction of astrocytes can be reversed by enhancing OPC‐astrocyte crosstalk and reinforcing their Wnt/*β*‐catenin signaling, implying a possible strategy for the intervention or treatments of parental isolation‐related neuropsychiatric diseases.

OPCs localize throughout the CNS and continuously differentiate into myelinating OLs from the early postnatal stages, which leads to difficulties in dissecting the role of OPCs from their myelinating descendants.^[^ ^40^ ^]^ In parental isolated mice, we chronologically traced the developmental changes in the early stages, and found that astrocytic development is impaired as early as P7 prior to OL differentiation/myelination. Moreover, specifically knocking out Wnt7b in OPCs resulted in astrocytic abnormalities and neuronal dysfunction in the hippocampus at an earlier stage as compared to OL differentiation. Though the connection of OL myelination to neuropsychiatric symptoms at the later stages of our models cannot be yet fully ruled out, our evidence demonstrates a novel Wnt paracrine‐dependent but myelination‐independent function of OPCs, which may provide a new therapeutic target for the onset of the early life stress‐related mental disorders.

## 4 Experimental Section

### Mice

All mice were housed in a temperature‐ and humidity‐controlled environments with free access to standard chow and water and on a 12 h/12 h light/dark cycle according to the guidelines of laboratory animal welfare and ethics committee of the Third Military Medical University. All mouse strains were maintained in the Third Military Medical University specific pathogen‐free animal facility.

The C57BL/6 wild‐type mice were purchased from the university animal breeding center. To establish the parental isolation model, isolation treatment was conducted between P2 to P12, where mouse pups were separated from their parents and housed individually for 8 h daily from 9:00 to 17:00, and were then returned to their parents from 17:00 to 9:00 (+1 day). To establish the Father‐only control group, the littermates were separated from the mothers but remain with their fathers during the separation period.

Pdgfra^CreER/+^ and DTA mice were described previously.^[^ ^24^, ^41^ ^]^ They were gifted by Dr. Stephen Fancy (University of California San Francisco), and crossed together to generate Pdgfra^CreER/+^:DTA/+ mice and the non‐creER:DTA/+ littermate control. To achieve OPC‐specific DTA expression and subsequent OPC elimination, tamoxifen (Sigma‐Aldrich, Cat: T5648, dissolved in 10% ethanol and 90% sunflower oil at 10 mg mL^−1^) was administrated via oral gavage daily from P4 to P7 at the dosage of 10 mg kg^−1^.

ROSA‐YFP reporter mice were described previously^[^ ^42^ ^]^ and were gifted by Dr. Stephen Fancy. These mice were crossed with the Pdgfra^CreER/+^ mice to validate the oligodendroglial‐specific targeting. Cre‐mediated recombination was induced using the same protocol as in the Pdgfra^CreER/+^:DTA/+ mice.

hGFAP^CreERT2^ mice were a generous gift from Dr. Christian Giaume (Collège de France) (MGI:4418665) and were described previously.^[^ ^43^ ^]^ mT/mG mice were obtained from the Jackson Laboratory (Cat: 007676) and were previously described.^[^ ^44^ ^]^ These two strains were mated to visualize astrocytic morphology after 3 consecutive days of daily tamoxifen treatment.

Wnt7a‐flox and the Wnt7b‐flox mice were newly generated using the CRISPR‐Cas9 system by the insertion of loxP sites flanking exon 3 and 4 of Wnt7a, and exon 4 of Wnt7b, respectively, into C57BL/6 mouse zygotes, which were transferred to pseudo‐pregnant CD1 mice at the blastocyst stage. These procedures were performed by Gempharmatech Co., Ltd. in China. The mice were then crossed with Pdgfra^CreER/+^ mice to generate Pdgfra^CreER/+^: Wnt7a‐flox/flox or Pdgfra^CreER/+^: Wnt7b‐flox/flox conditional knockout (KO) mice. Non‐CreER: Wnt7a‐flox/flox or non‐CreER: Wnt7b‐flox/flox littermates were used as the control group.

### Primary Cell Cultures

Primary astrocytes were isolated from P1‐2 mouse brains as previously described.^[^ ^45^ ^]^ Cells were cultured in Dulbecco's modified Eagle medium (DMEM) with 10% fetal bovine serum (FBS) and plated on poly‐L‐lysine‐coated coverslips.

The mouse OPCs were prepared by immunopanning from P7 mouse cortices as previously described.^[^ ^46^ ^]^ Briefly, panning dishes were incubated with secondary and primary antibodies sequentially. Mouse brain cerebral hemispheres were diced and dissociated using papain at 37 °C for 75 min. After trituration, cells were resuspended in a panning buffer and then incubated at room temperature sequentially on three immunopanning dishes: Ran‐2, GalC, and PdgfaR. The isolated OPCs were then cultured in the OPC‐proliferation medium on poly‐D‐lysine‐coated plates.

For astrocyte‐OPC co‐culture, the purified OPCs (4.5 × 10^4^ cells per well) were seeded onto primary astrocytes in 24‐well plates and maintained in OPC‐proliferation medium.

For in vitro endothelial cell experiments, primary cultured rat endothelial cells were isolated from P14 SD rats as previously described,^[^ ^17^ ^]^ and cultured on collagen type I and fibronectin pre‐coated coverslips for immunostaining. Conditioned medium (75% fresh medium + 25% conditioned medium) and exogenous Wnt ligand treatment experiments were conducted by culturing cells for 24 h.

### Behavioral Tests

All mice were kept in a controlled environment (25 °C) with free access to food and water, and housed on a 12 h/12 h light/dark cycle. All tests were performed from 12:00 to 18:00. After each trial, all apparatus were wiped with 30% ethanol to remove residual smell cues. For all behavioral experiments, investigators were blinded to genotype, and care was taken to handle the mice gently to avoid stress. At least eight mice were included in each experimental group in the behavioral tests.

*Cliff Avoidance Reaction Test*: This experiment was performed to evaluate the impulsive behavior as described previously.^[^^47^^]^ Briefly, the cliff avoidance reaction (CAR) was assessed using a round plastic platform (diameter of 16 cm; thickness of 2 cm), supported by an iron rod (height of 50 cm). The platform was secured such that animal movement did not affect platform stability. The floor below the platform was carpeted to prevent injury from the animal fall. The test was initiated by gently placing a single animal on the platform. Latency from the initial placement on the platform until falling and the incidence of falling were recorded. The incidence of impaired CAR was calculated as a percentage index for each group: %(CAR)  =  the number of intact CAR mice (which did not fall from platforms)/total numbers of tested mice × 100. The end of the test was determined when the mice fell from the platform or when a total of 1200 s had elapsed.

*Elevated Plus Maze Test*: The elevated plus maze test was used to evaluate anxiety‐related behavior in the mice as previously described.^[^ ^48^ ^]^ The mouse was placed in the elevated plus maze with two opposing closed arms and two opposing open arms. Mouse activity was recorded for a total of 10 min. The number of entries into the open arm and the time spent in the open arm were measured.

*Tail Suspension Test*: The tail suspension test was used to study depression‐associated behavior as described previously.^[^ ^49^ ^]^ Briefly, the mice were suspended by sticking the end of the tail to the edge of a shelf using adhesive tape. Mouse activity was recorded, and immobility time was measured. The test was continued for a total of 6 min.

*Open Field Test*: The experiment was performed using an open field activity system (Biowill, Shanghai, China). Briefly, mice were placed in the center of an open field box (50 × 50 × 50 cm), and their activity was recorded for 20 min. The time and distances traveled in the center zone, as well as the total distances traveled, were measured.

*Novel Object Recognition Test*: The experiment was conducted as described previously.^[^^50^^]^ Briefly, a white polyvinyl plastic square open field (25 cm × 25 cm × 40 cm) with two rectangular plastic objects placed 8 cm away from the walls was set up. The mouse was introduced into the apparatus for 5 min. 2 h later, one of the rectangular objects was replaced by a cylinder (a new toy/novel object) before the mouse was re‐introduced into the apparatus. The discrimination index was determined to be the ratio of the time spent exploring the novel object to the total time spent exploring both objects.

### Osmotic Pump Implantation

Osmotic pumps and catheters were purchased from ALZET (0004317 micro‐osmotic pump model 1002; 0008851 brain infusion kit 3, 1–3 mm) to deliver Wnt ligands directly into the mouse hippocampus. The micro‐osmotic pump was connected to the catheter, filled with 150–200 µL of saline spiked with (or without) Wnt ligands at a fixed concentration (Wnt7a: 50 ng mL^−1^; Wnt7b: 50 ng mL^−1^), then implanted subcutaneously following the manufacturer's instructions (ALZET) in concurrence with previous studies.^[^ ^51^ ^]^ The stereotaxic coordinates for P14 mouse implantation used were as follows: 2 mm cephalic to Lambda, 1.0 mm lateral to Lambda, and 2.0 mm deep in the dorsoventral direction. At P18 (after a 4 day infusion), the pump was removed after the mouse was transcardially perfused with 4% formaldehyde.

### Immunohistochemistry

The method was described previously.^[^ ^17^ ^]^ Primary antibodies used were: Rabbit anti‐NeuN (Abcam, ab177487), Rabbit anti‐c‐Fos (CST, 2250S), Rabbit anti‐BLBP (Abcam, ab32423), Goat anti‐GFAP (Abcam, ab53554), Rabbit anti‐Cx43 (Thermo, 710700), Rabbit anti‐Cx30 (Abcam, ab200866), Rabbit anti‐NG2 (Millipore, MAB5320), Rat anti‐PDGFR*α* (BD Biosciences, 558774), Mouse anti‐Olig2 (Millipore, MABN50), Rabbit anti‐Olig2 (Millipore, AB9610), Mouse anti‐CC1 (Millipore, OP80), Rat anti‐MBP (Millipore, MAB386), Rabbit anti‐Iba1 (Wako, 019–19741), Rabbit anti‐Ki67 (Thermo, MA514520), Goat anti‐GFP (Abcam, ab5450), Rat anti‐CD31 (BD Biosciences, 553370), Rat anti‐PDGFR*β* (Invitrogen, 14‐1402‐81), Rabbit anti‐Wnt7a (Abcam, ab100792), Rabbit anti‐non‐phospho (Active) *β*‐Catenin (CST, 8814S). Images were captured using VS200 Research Slide Scanner (Olympus), FV3000 confocal microscope (Olympus), or Axio Imager M2 with the apotome system (Zeiss).

### TUNEL Assays

To assay for apoptosis, the TUNEL (terminal deoxynucleotidyl transferase dUTP nick end labeling) assay was performed following the manufacturer's instructions (Roche, in situ cell death detection fluorescein, 11684795910). The specimens were then subjected to immunofluorescence staining of NG2. The specimens were observed using a fluorescence microscope at an excitation wavelength in the range of 450–500 nm.

### Acute Brain Slice Preparation

Acute brain slices were prepared as described previously.^[^ ^45^ ^]^ Briefly, brain slices were cut on a vibratome (Leica, VT1200) and transferred to a holding chamber where they were rested on a nylon mesh, submerged in oxygenated artificial cerebrospinal fluid (ACSF) for a stabilization period of 60 min prior to further studies.

### Calcium Imaging

The method has been described previously.^[^ ^52^ ^]^ Briefly, acute brain slices were prepared as described above and then incubated in Rhod‐4 (abcam, Cat: ab112157) following the manufacturer's instructions for examining intracellular calcium assays. The slices were incubated in Rhod‐4 working solution (500 *μ*L in 2 mL ACSF) supplied with 95% O_2_/5% CO_2,_ in the dark at 37 °C for 30 min, followed by 30 min at room temperature before being transferred to the recording chamber. For imaging and quantification of intracellular calcium waves, confocal images were acquired using an FV1000 Olympus microscope. Slices were placed in a perfusion chamber on the stage of the confocal microscope and perfused with oxygenated ACSF. The calcium imaging experiments were divided into two phases. During the initial phase, baseline fluorescence was collected every 3.45 s for 150 s. In the second phase, the slices were subsequently perfused with oxygenated ACSF, and the fluorescence signal was recorded for another 350 s.

For calcium imaging in primary astrocytes, the cells were incubated with Fluo‐4 in the dark at 37 °C for 30 min before being transferred to the recording chamber. For imaging and quantification of intracellular calcium waves, confocal images were acquired using an FV1000 Olympus microscope. The calcium imaging experiments were consisted of two phases. During the initial phase, the baseline fluorescence was collected every 3.6 s for 300 s, followed by the second phase whereupon KCl (20 × 10^−6^ m) was added to the culture, and fluorescence was collected for another 350 s. Data were analyzed using the cellSens Software (Olympus, Japan).

### gap‐FRAP Assay

Acute brain hemisphere slices were prepared as described above and then were incubated with ACSF containing SR101 (1 × 10^−6^ m) at 37 °C for 30 min. After a 20 min wash in ACSF, slices were placed in a perfusion chamber on the stage of the confocal microscope (FV3000, Olympus) and perfused with oxygenated ACSF during the entire experiment. gap‐FRAP experiments were consisted of a three‐part illumination sequence. During the initial part, the baseline fluorescence was collected every 5 s for 15 cycles on a 158 × 158 µm field, with a low level of laser power to avoid photobleaching. Next, in order to photobleach the cell body of the target astrocyte, the laser scanning area was restricted to a smaller field (15 × 15 µm), whereupon 561 nm laser light was applied at a power of 550 V (80%) for 20 s. Then, the fluorescence recovery was recorded every 5 s during the subsequent 500 s. Images were analyzed offline: the mean fluorescence intensity (F0) of the astrocyte cell body was measured using the cellSens Software (Olympus, Japan). F0 was obtained from the first value of the baseline recording. The fluorescence intensity of each time point was normalized to F0 and then expressed as the percentage of F0 (Ft/F0%). The percentage of recovery was calculated by using the plateau value (the average of the last six values of the recording) subtracting the value of the first time point after photobleaching. All studies were performed in the CA1 region of the hippocampus at a depth of 25 µm below the surface to ensure that the cells of interest were not been damaged during slicing.

### OPC/OL Conditioned Medium and Membrane Protein Collection

The purified OPCs (6 × 10^5^ cells per well) were plated in coated six‐well plates in 2 mL OPC‐proliferation medium. The OPC‐conditioned medium was collected 48 h later and replaced with an OPC‐differentiation medium. The medium was collected after 48 h as the pre‐OL conditioned medium, with new OPC‐differentiation medium replenished to the cells. The medium was then collected after 48 h as the OL‐conditioned medium. The purified OPCs and OLs in coated 6 cm dishes were scraped at the indicated time points and disrupted by repeated freeze–thaw cycles. The cell pellet was washed with pre‐cold DMEM twice, resuspended in DMEM, and homogenized using a probe sonicator on ice.

### Scratch Loading and Dye Transferring Assay

For functional gap junction evaluation in vitro, the scratch assay was conducted as previously described.^[^ ^53^ ^]^ Briefly, astrocyte cultures were grown to confluency before the assay. Cells were rinsed with CaMg‐PBS (phosphate‐buffered saline). 1 mg mL^−1^ solution of Lucifer yellow dye (L0259; Sigma) was added to cover the cell monolayer, and the cells were scraped using a surgical blade. After 1 min incubation at room temperature, the cells were washed thoroughly with PBS and re‐incubated with culture medium, then observed under a fluorescence microscope.

### EdU Assay

Primary cultured endothelial cells were treated with OPC‐CM, OL‐CM, and Wnt ligands. XAV939 was used as the Wnt pathway inhibitor. Endothelial cells were cultured with those conditions for 24 h for the proliferation assay. To label the dividing endothelial cells, 5‐ethynyl‐2’‐deoxyuridine (50 × 10^−6^ m) was diluted 1:1000 in culture medium and added to wells for 1 h incubation. Then endothelial cells were fixed for 15 min in 4% paraformaldehyde and stained using the EdU Imaging Kit (Ribobio, C10310‐3).

### RNA‐seq

RNA‐seq experiments were performed using freshly isolated astrocytes and OPCs from the Father‐only group and the Isolated group at P7. Astrocytes and OPCs were isolated by immunopanning as described previously.^[^ ^46^, ^54^ ^]^ RNA was extracted from the isolated cells by Trizol (Thermo) according to the manufacturer's protocol. RNA‐seq was performed by the Beijing Genomics Institute (BGI). Differential expression analysis was performed on read counts using DESeq2. Pair‐wise comparisons were performed between the Father‐only and the isolated littermates. Differentially expressed genes were selected by using a significance threshold of FDR < 0.05, log2FC > 0.26 or log2FC < −0.26, and subjected to pathway enrichment analysis using DAVID (https://david.ncifcrf.gov/).

### Enzyme‐Linked Immunosorbent Assay (ELISA)

To examine the concentrations of Wnt3a, Wnt7a, and Wnt7b proteins in OPC/OL conditional medium and brain lysates, ELISA kits specific to the ligands (mouse Wnt3a Elisa Kit, CSB‐EL026136MO; mouse Wnt7a Elisa Kit, CSB‐EL026141MO; mouse Wnt7b Elisa Kit, CSB‐EL026142MO; CUSABIO) were used according to the manufacturer's instructions. The OD values were determined by measuring the absorbance at 450 nm using a microplate reader (Bio‐RAD, Model 680). Independent experiments were performed in triplicate.

### RT‐qPCR

Total ribonucleic acid (RNA) was isolated using RNeasy Plus Mini Kit (Qiagen, 74134). Quantitative polymerase chain reaction (qPCR) was performed with the Real‐time PCR Detection System (Roche) and FastStart Universal SYBR Green Master (Roche, 04913850001). Amplification and melt curve analysis was performed. For each sample, independent repeats were performed in triplicate.

### Western Blot

Sodium dodecyl sulfate–polyacrylamide gel electrophoresis (SDS‐PAGE) Western blot was carried out to quantify Cx43, GFAP protein level changes. Protein samples were separated on 10% SDS‐PAGE gels, transferred to nitrocellulose membranes and probed with antibody against Cx43 (Rabbit, Thermo, 710700), GFAP (Rabbit, Abcam, ab7260). *β*‐actin was used as protein loading control (Mouse, Beyotime, AF0003). Protein bands were visualized by chemiluminescence (ECL Plus, GE Healthcare) after incubation with HRP‐conjugated secondary antibodies. Images were captured by ChampChemi 610 Chemiluminescence Imaging System (Beijing Sage Creation, China). Band intensity was quantified using the Image Pro Plus image analysis system.

### Quantification Methods

Images captured with the Olympus VS200 Research Slide Scanner were used to quantify cell numbers. The number of cells in a fixed area in the CA1 region was quantified manually. Quantification of Cx43 and Cx30 area, or the number of cell branches was done using images captured by the Zeiss Axio Imager M2, which were then analyzed using the Fiji software. For quantification of immunohistochemistry of mouse samples, at least three brain slices were analyzed for each mouse, and three mice were analyzed for each experimental group. For quantification of cell culture, three coverslip repeats were done for each treatment group, while at least five images from each coverslip were analyzed.

### Statistical Analysis

Statistical significance between groups was determined using the GraphPad Prism software 9.0 (GraphPad Software, San Diego, CA, USA). Data collected were normalized to the control group. All graph data were presented as the mean ± SD unless specified otherwise. For behavioral experiment, *N* ≥ 7 mice. For immunohistochemistry, RT‐qPCR, Western blot, and RNA‐seq experiments, *N* = 3 mice. For primary cell culture experiments, N = 3 independent experiments. Data for gap‐FRAP (*n* ≥ 10 cells) and calcium imaging experiment (*n* ≥ 27 cells) were pooled from three independent experiments. The sample size per group was determined in accordance with previous publication with similar methologies. No data were excluded from statistical analysis. Data normality was accessed by the Shapiro–Wilk test. The unpaired *t*‐test was used to determine the significance between two experimental groups, while one‐way analysis of variance (ANOVA) was used to determine statistical significance when comparing multiple groups. All statistical tests were two‐tailed. *p*‐values less than 0.05 were considered statistically significant. Significant statistical results were indicated as: *p < 0.05, **p < 0.01, ***p < 0.001, ****p < 0.0001.

## Conflict of Interest

The authors declare no conflict of interest.

## Author Contributions

Y.X.W. and Y.X.S. contributed equally to this work. Conceptualization, J.Q.N.; Methodology, C.J.Y. and J.Q.N.; Investigation, Y.X.W., Y.X.S., X.R.W., G.D.Y., Y.J.C., and R.L.; Visualization, Y.X.W., Y.X.S., and J.Q.N.; Writing—Original Draft, Y.X.S. and J.Q.N.; Writing—Review & Editing, C.J.Y., L.X., and J.C.S.; Resources, X.Y.C. and B.Y.; Funding Acquisition, C.J.Y., L.X., J.C.S. and J.Q.N.; Supervision, C.J.Y., L.X., and J.Q.N.

## Supporting information

Supporting Information

## Data Availability

The data that support the findings of this study are available from the corresponding author upon reasonable request.
