# Supplementary material for: Reduced Oligodendrocyte Precursor Cell Impairs Astrocytic Development in Early Life Stress
Source: Adv Sci (Weinh). 2021 Jun 21;8(16):2101181. doi: 10.1002/advs.202101181 (PMC8373108; doi:10.1002/advs.202101181)
Supplement: Supplementary file 1 — Supporting Information [file ADVS-8-2101181-s001.pdf]

© 2021 Wiley-VCH GmbH

ADVANCED SCIENCE
Open Access

Supporting Information

for *Adv. Sci.*, DOI: 10.1002/advs.202101181

# Reduced oligodendrocyte precursor cell impairs astrocytic development in early life stress

*Yuxin Wang, Yixun Su, Guangdan Yu, Xiaorui Wang, Xiaoying Chen, Bin Yu, Yijun Chen, Rui Li, Juan C. Sáez, Chenju Yi*, Lan Xiao*, Jianqin Niu**

# Supporting Information

**Title: Reduced oligodendrocyte precursor cell impairs astrocytic development in early life stress**

*Yuxin Wang[#], Yixun Su[#], Guangdan Yu, Xiaorui Wang, Xiaoying Chen, Bin Yu, Yijun Chen,*

*Rui Li, Juan C. Sáez, Chenju Yi*, Lan Xiao*, Jianqin Niu**

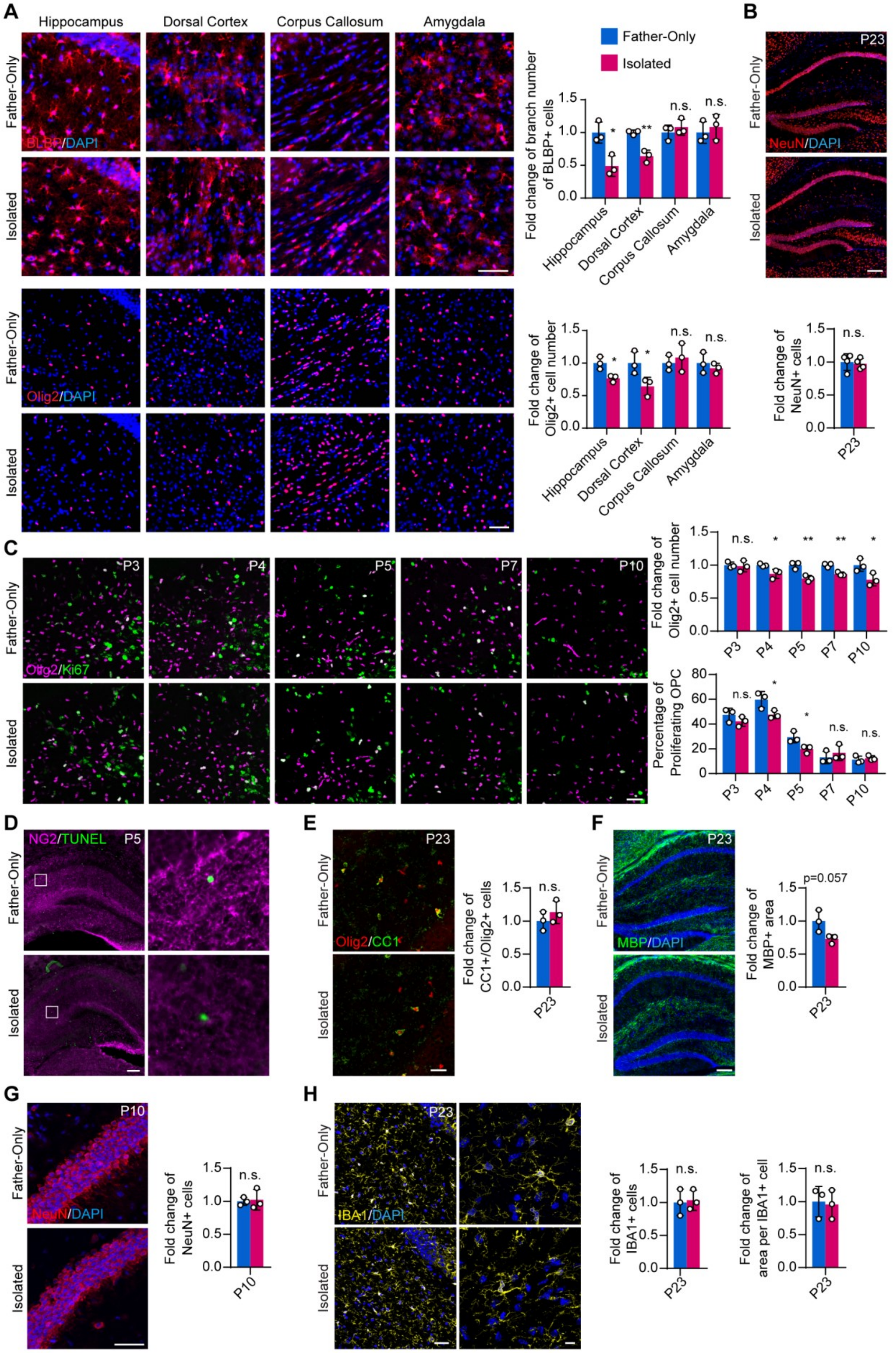
A
Hippocampus
Dorsal Cortex
Corpus Callosum
Amygdala
Father-Only
Isolated
BLBP/DAPI
Olig2/DAPI
Fold change of branch number of BLBP+ cells
Fold change of Olig2+ cell number
n.s.
B
P23
NeuN/DAPI
Fold change of NeuN+ cells
C
P3
P4
P5
P7
P10
Olig2/Ki67
Percentage of Proliferating OPC
D
NG2/TUNEL
E
Olig2/CC1
Fold change of CC1+/Olig2+ cells
F
MBP/DAPI
p=0.057
Fold change of MBP+ area
G
H
IBA1/DAPI
Fold change of IBA1+ cells
Fold change of area per IBA1+ cell

**Figure S1. Histological characterization of the isolation model. Related to Figure 1 and Figure 2.**

**(A)** Upper panel: BLBP staining showed that shrunken astrocyte morphology could be found in the hippocampus and the dorsal cortex but not in the corpus callosum or the amygdala of the isolated mice at P23. Lower panel: Olig2 staining showed that alteration of OPC numbers could be found in the hippocampus and the dorsal cortex but not the corpus callosum or the amygdala of isolated mice at P23. Scale bar: 50 µm. **(B)** The isolation group did not show a reduction of $NeuN^+$ neuron numbers in the hippocampus. Scale bar: 100 µm. **(C)** Immunostaining of Ki67 and Olig2 in P3-P10 hippocampus of the isolation model. Scale bar: 50 µm. The number of $Olig2^+$ cells started to decrease in the isolated mice at P4. Similarly, the percentage of the proliferating OPC ($Ki67^+Olig2^+/Olig2^+$) was also reduced in the isolated mice at P4 and P5. **(D)** TUNEL assay followed by immunostaining of NG2, showing no difference in OPC death between the two groups. Scale bar: 100 µm. **(E)** Immunostaining of Olig2 and CC1 showed that the ratio between $CC1^+$ cells and $Olig2^+$ cells was unaltered. Scale bar: 20 µm. **(F)** The isolated group displayed a slight but non-statistically significant decrease in the $MBP^+$ areas in the hippocampus. Scale bar: 100 µm. **(G)** The number of $NeuN^+$ neurons was not altered in the isolated group at P10. Scale bar: 50 µm. **(H)** There were no significant differences in the $IBA1^+$ microglia number and morphology between the Father-only and the isolated group. Scale bar: 50 µm (Left) or 10 µm (Right).

Data presented as mean ± SD. N=3 mice for immunostaining experiments. *p*-values are calculated using unpaired t-test. * $p < 0.05$, ** $p < 0.01$, n.s. not significant.

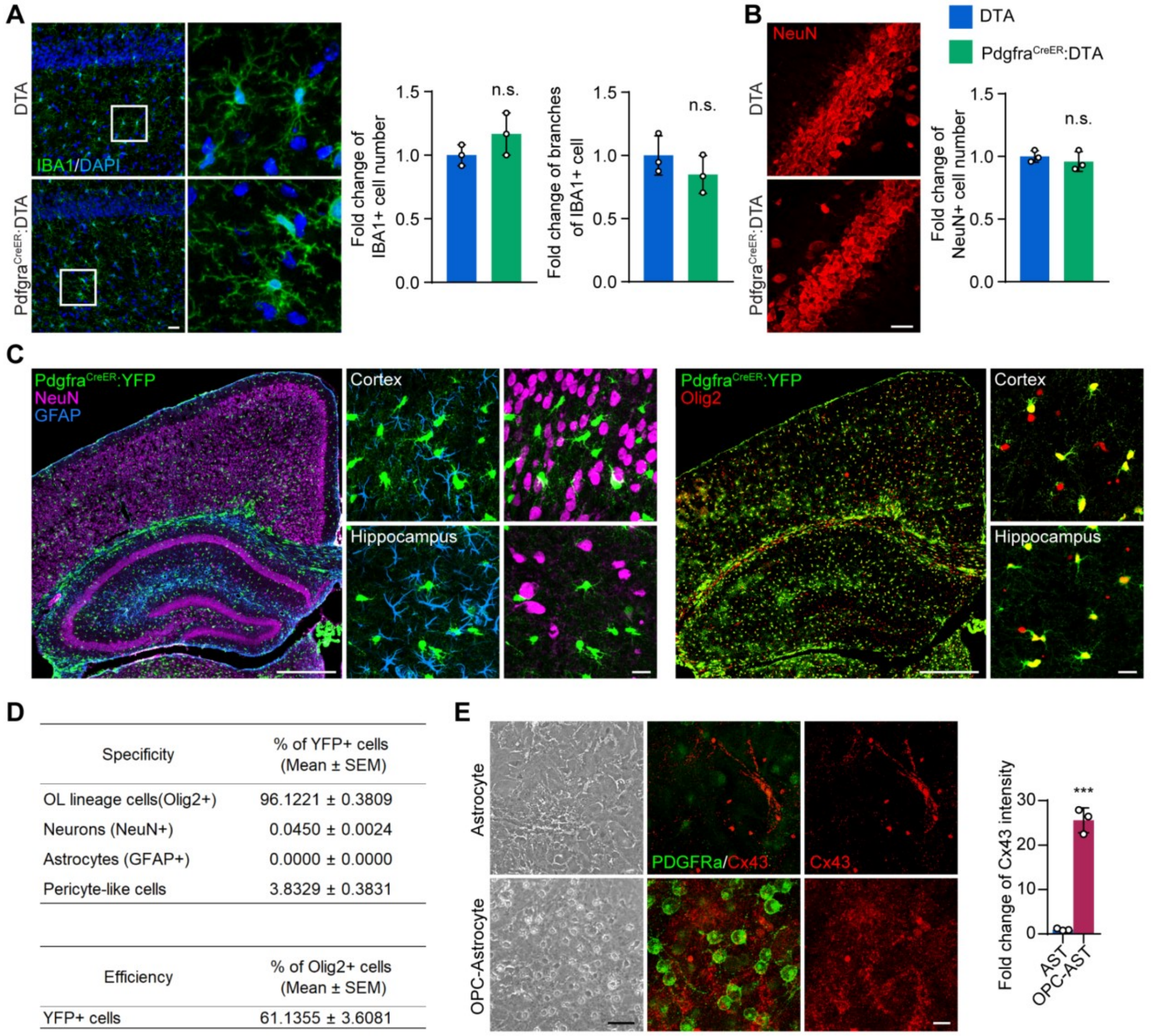


**Figure S2. Reduced OPC population causes astrocytic network dysfunction. Related to Figure 3.**

**(A)** Immunostaining of IBA1 showed no differences in microglia number and morphology in the hippocampus. Scale bar: 50 μm. **(B)** Immunostaining of NeuN showed no difference in hippocampal neuron number. Scale bar: 20 μm. n.s. not significant (N=3 mice). **(C-D)** Rosa-YFP reporter mice were crossed with Pdgfra$^{CreER}$ mice to validate the specific oligodendroglial lineage targeting of the Pdgfra$^{CreER}$ mouse strain. 96.1221% YFP$^{+}$ cells were oligodendroglial lineage marker Olig2$^{+}$ in the whole coronal brain section. No astrocytes (GFAP$^{+}$) were found co-labled with Cre-driven YFP expression. 0.045% YFP$^{+}$ cells were neuronal marker NeuN+ in the whole coronal brain section, while 3.8329% YFP$^{+}$ cells were Olig2- and displayed

pericyte-like morphology. In addition, Pdgfra$^{CreER}$ mice achieved recombination in 61.1355% Olig2$^{+}$ cells. Scale bar: 500 μm or 20 μm. **(E)** Immunostaining of PDGFRa and Cx43 in astrocytes co-cultured with OPC. Scale bar in brightfield: 50 μm, in immunofluorescence: 10 μm.

Data presented as mean ± SD. N=3 mice or 3 independent experiments. *p*-values are calculated using unpaired t-test. *** $p < 0.001$, n.s. not significant.

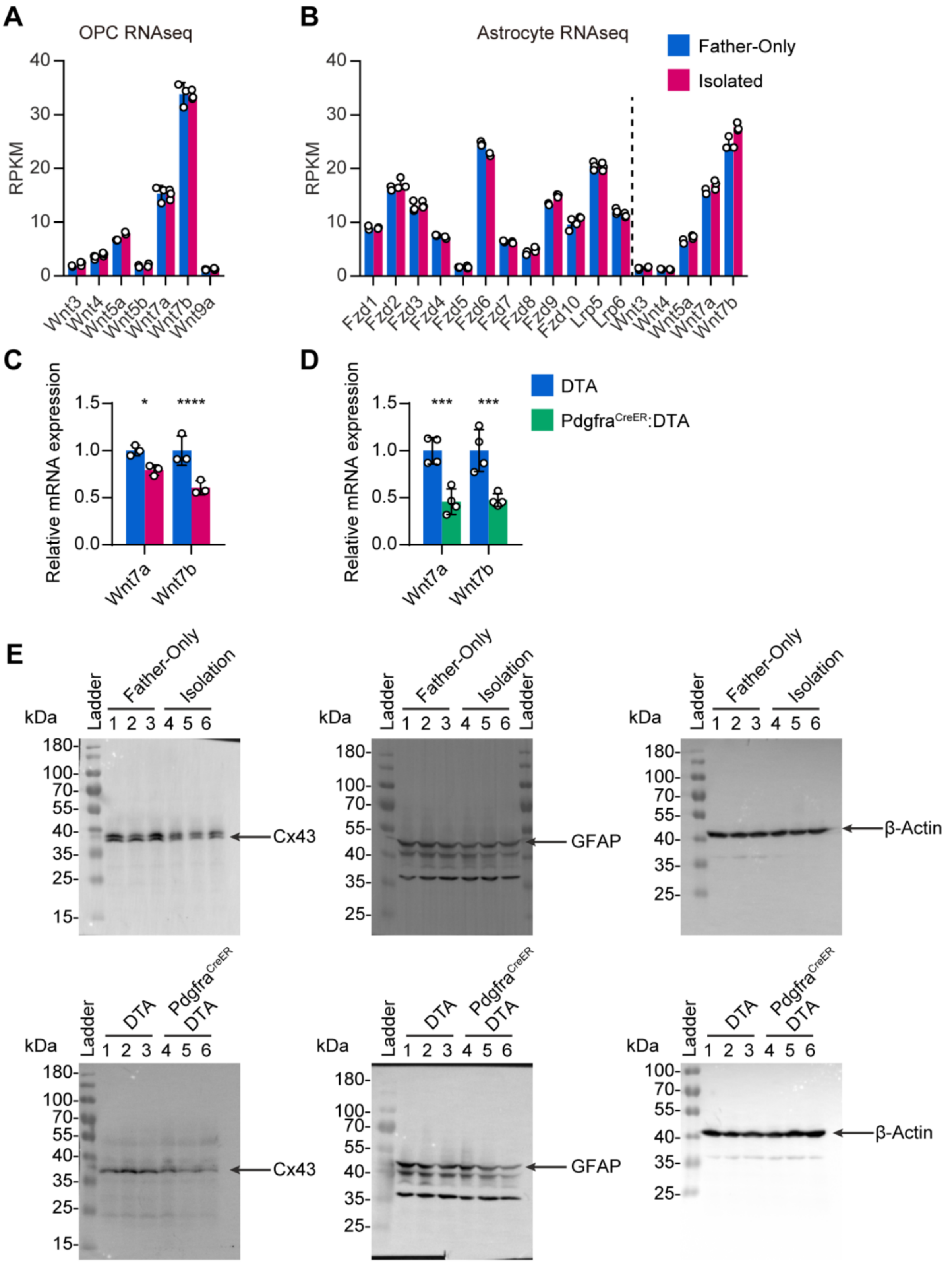


**Figure S3. OPCs regulate the astrocyte network through secreted Wnt7a/b. Related to Figure 4 and Figure 5.**

**(A)** RNA-seq of OPC showed that Wnt7a and Wnt7b are highly expressed in OPC and Wnt ligand expressions were not significantly altered in the parental isolation model. **(B)** Expression of Wnt receptors and ligands in astrocytes extracted from RNA-seq data. **(C-D)** RT-qPCR conducted on brain lysates revealed decreased expression of Wnt7a and Wnt7b in isolated mice and OPC-ablated mice compared to controls. **(E)** The full western blot images overlayed with the membrane in **Figures 4H and 4J**.

Data presented as mean ± SD. N=3 mice in maternal isolation model, N=4 mice in OPC-ablated mouse model. *p*-values are calculated using unpaired t-test. * $p < 0.05$, *** $p < 0.001$, **** $p < 0.0001$.

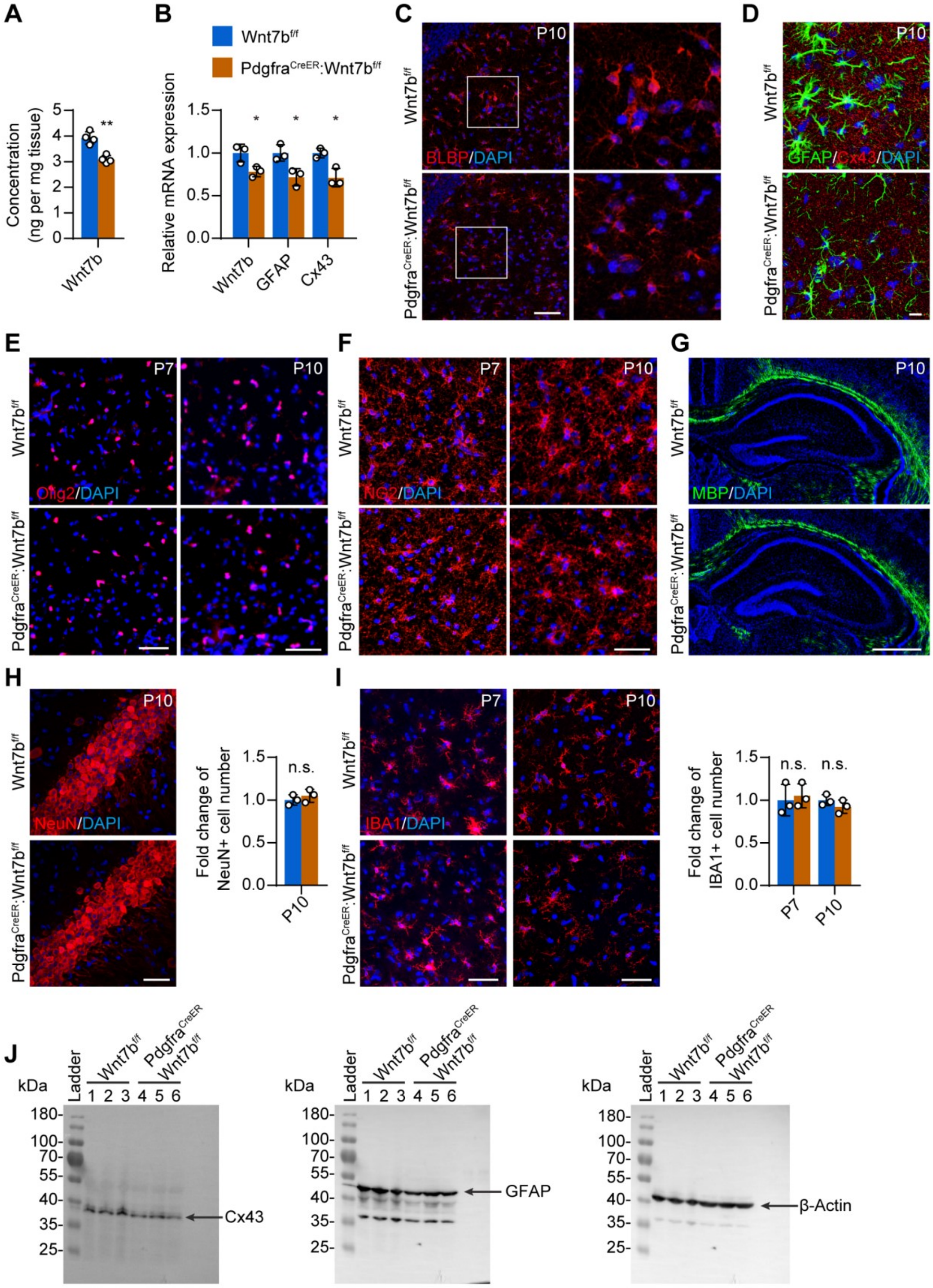


**Figure S4. Characterization of Wnt7b cKO mice. Related to Figure 6.**

**(A)** ELISA assay of Wnt7b on brain lysates from Wnt7b cKO and littermate control at P10. **(B)** qPCR experiments on brain lysates of Wnt7b cKO and littermate controls at P10. **(C)** Immunostaining of BLBP at P10. Scale bar: 50 µm. **(D)** Immunostaining of GFAP and Cx43 at P10. Scale bar: 5 µm. **(E)** Immunostaining of Olig2 at P7 and P10. Scale bar: 50 µm. **(F)** Immunostaining of NG2 at P7 and P10. Scale bar: 50 µm. **(G)** Immunostaining of MBP showed that no myelination took place in the hippocampus at P10 in both control and Wnt7b cKO mice. Scale bar: 500 µm. **(H)** Wnt7b cKO did not alter the $NeuN^+$ neuron number in the hippocampus at P10. Scale bar: 50 µm. **(I)** Wnt7b cKO did not affect the $IBA1^+$ microglia number in the hippocampus at P7 and P10. Scale bar: 50 µm. **(J)** The full western blot images overlayed with the membranes in **Figure 6D**.

Data presented as mean ± SD. N=3 mice. *p*-values are calculated using unpaired t-test. * $p < 0.05$, ** $p < 0.01$, *** $p < 0.001$, n.s. not significant.

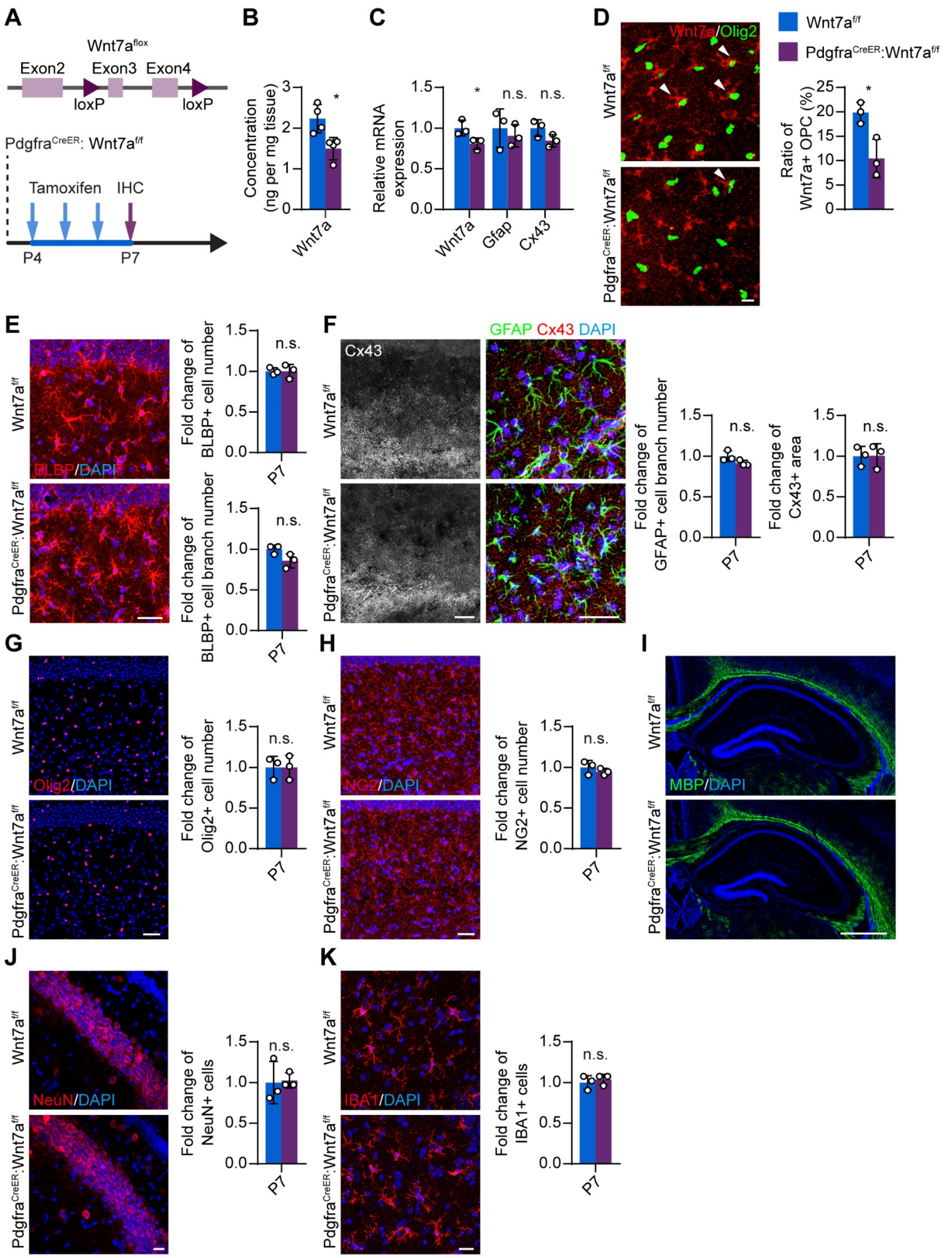


**Figure S5. Wnt7a conditional knockout in OPC did not have a significant effect on astrocytes, OPCs and neurons at P7. Related to Figure 6.**

**(A)** Construction of Wnt7a-floxed mouse strain and the overall experiment scheme. **(B)** ELISA assay of Wnt7a on brain lysates from Wnt7a cKO and the littermate control at P7. **(C)** qPCR experiments on brain lysates of Wnt7a cKO and the littermate controls at P7. **(D)** Immunostaining of Wnt7a and Olig2 showed that Wnt7a cKO achieved Wnt7a conditional knockout in $Olig2^+$ cells. White arrowheads highlight the $Wnt7a^+$:$Olig2^+$ cells. **(E)** Immunostaining of BLBP and quantification of $BLBP^+$ cell number as well as the cell process number. Scale bar: 50 µm. **(F)** Immunostaining of GFAP and Cx43. The branch number of $GFAP^+$ cells, and Cx43 intensity were quantified. Left scale bar: 50 µm. **(G)** Immunostaining of Olig2 reflected no significant change in the Olig2+ OPC number in the Wnt7a cKO. Scale bar: 50 µm. **(H)** Immunostaining of NG2 found no significant change in the $NG2^+$ OPC population in the Wnt7a cKO. Scale bar: 50 µm. **(I)** Immunostaining of MBP showed that no myelination took place in the hippocampus at P7 in both control and Wnt7a cKO mice. Scale bar: 500 µm. **(J)** Wnt7a cKO did not alter the $NeuN^+$ neuron number in the hippocampus. Scale bar: 20 µm. **(K)** Wnt7a cKO did not affect the hippocampal $IBA1^+$ microglia number. Scale bar: 20 µm.

Data presented as mean ± SD. N=3 mice. *p*-values are calculated using unpaired t-test. * $p < 0.05$, n.s. not significant.

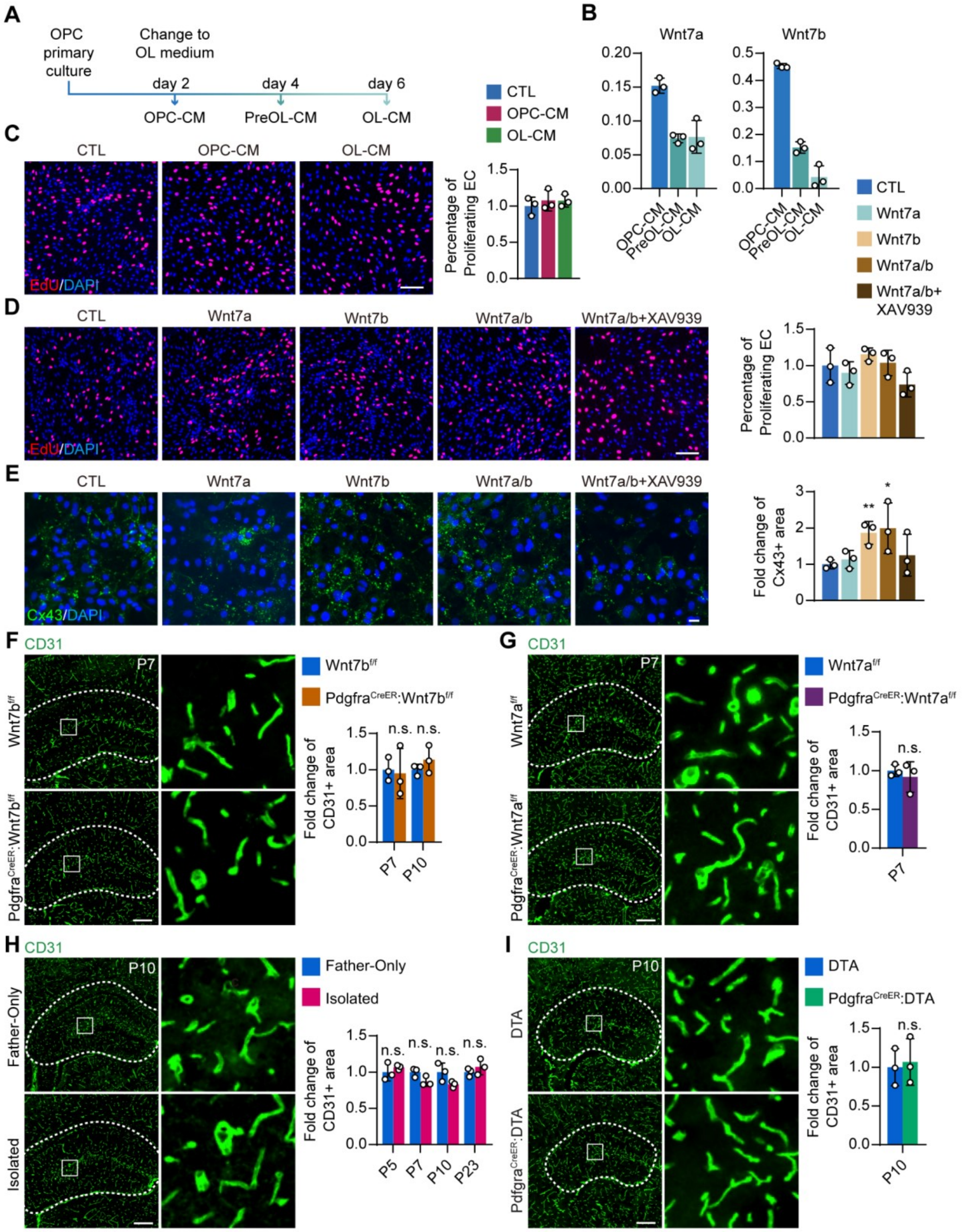


**Figure S6. Effect of OPC-CM and OPC-derived ligands on endothelial cells. Related to Figure 6.**

**(A)** Diagram describing the collection of OPC-CM, PreOL-CM, and OL-CM. **(B)** ELISA assay to determine the level of Wnt3a, Wnt7a and Wnt7b in the OPC-, PreOL- and OL-CM. **(C)**

Primary rat endothelial cells were treated with OPC-CM and OL-CM followed by EdU incorporation assay to examine the proliferation rate. OPC-CM and OL-CM treatment did not alter the proliferation of endothelial cells *in vitro*. Scale bar: 50 µm. **(D)** Comparably low levels of exogenous Wnt ligands as found in OPC-CM were applied to primary rat endothelial cells, followed by EdU incorporation assay to examine the proliferation rate. None of the treatments was able to induce a significant change in proliferation rate. Scale bar: 50 µm. **(E)** Comparably low levels of exogenous Wnt ligands as found in OPC-CM were applied to primary astrocytes, followed by immunostaining of Cx43. Wnt7b treatment, as well as Wnt7a+Wnt7b treatment, were able to induce Cx43 expression at such a low level. Scale bar: 5 µm. * $p<0.05$, ** $p<0.01$, n.s. not significant (N=3 experiments). Error bars represent SD. **(F)** Endothelial cells were labeled by CD31 antibody and the positive area in the hippocampus was quantified. Wnt7b cKO did not lead to change in $CD31^+$ area in the hippocampus at P7. Scale bar: 500 µm. **(G)** Wnt7a cKO did not alter the $CD31^+$ area at P7 and P10. Scale bar: 500 µm. **(H)** Parental isolation did not significantly alter the $CD31^+$ area from P5 to P23. Scale bar: 500 µm. **(I)** OPC-ablation did not result in $CD31^+$ area changes. Scale bar: 500 µm. The dashed line highlighted the hippocampus region.

Data presented as mean ± SD. N=3 mice. *p*-values are calculated using unpaired t-test. n.s. not significant.

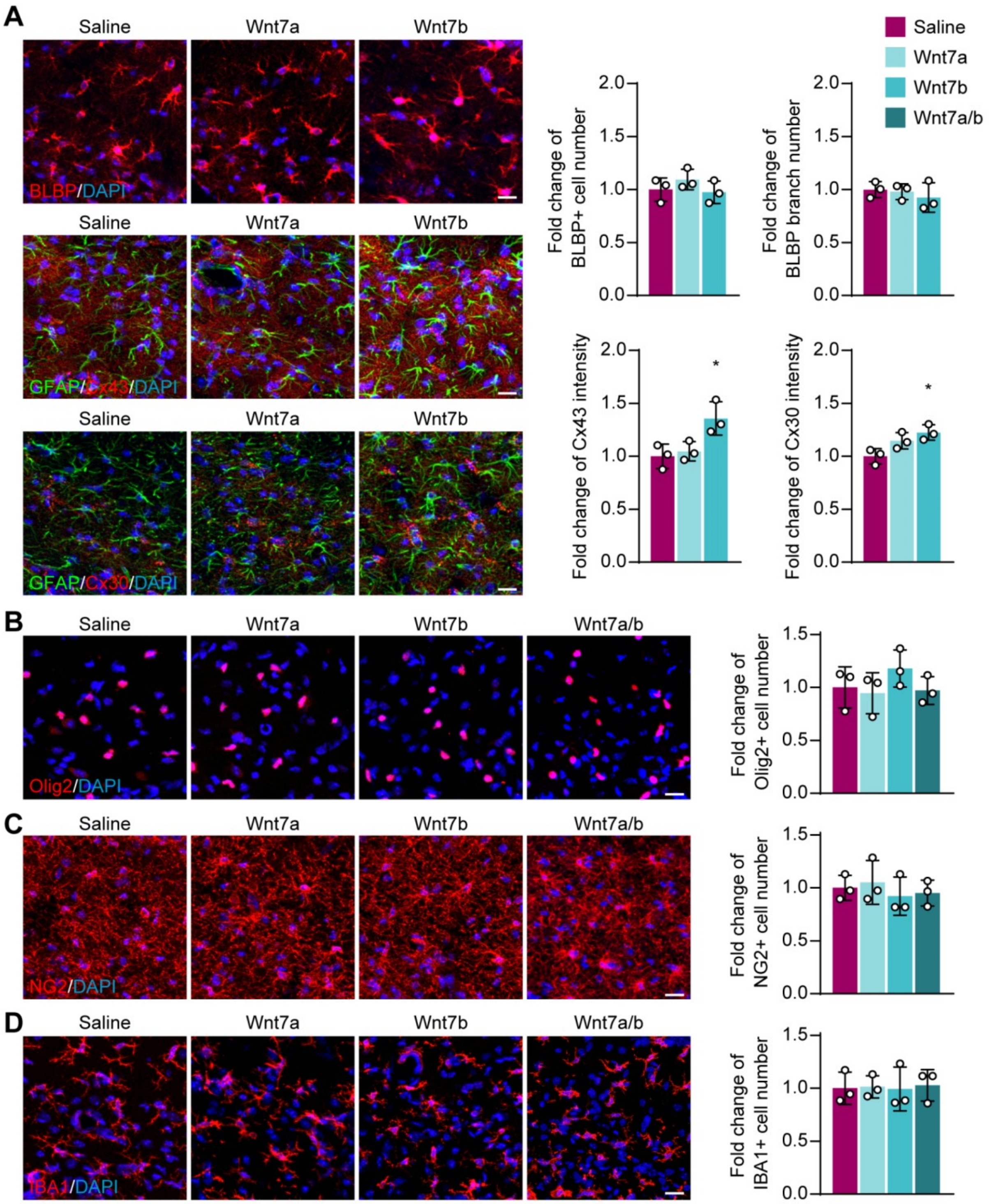


**Figure S7. Wnt7a and Wnt7b microinjection. Related to Figure 7.**

**(A)** micro-injection of only Wnt7a or Wnt7b in isolated mice followed by immunostaining of astrocyte markers. Wnt7a or Wnt7b microinjection did not significantly affect the BLBP+ cell number or branch number. However, Wnt7b microinjection but not Wnt7a was able to promote the expression of Cx43 and Cx30. **(B)** Immunostaining of Olig2 reflected no changes in Olig2$^{+}$

cell numbers in neither Wnt7a, Wnt7b, or co-injected group. Scale bar: 50 µm. **(C)** Immunostaining of NG2 showed that the $NG2^+$ OPC number was not altered by Wnt7a, Wnt7b injection or Wnt7a/b co-injection. Scale bar: 50 µm. **(D)** Immunostaining of IBA1 showed that the microglia number was not altered by Wnt7a, Wnt7b injection or Wnt7a/b co-injection. Scale bar: 20 µm.

Data presented as mean ± SD. N=3 mice. *p*-values are calculated using unpaired t-test. * $p < 0.05$.
